# Inhibition of MTA1 by ERα contributes to protection hepatocellular carcinoma from tumor proliferation and metastasis

**DOI:** 10.1186/s13046-015-0248-0

**Published:** 2015-10-26

**Authors:** Lei Deng, Hui Yang, Junwei Tang, Zhe Lin, Aihong Yin, Yun Gao, Xuehao Wang, Runqiu Jiang, Beicheng Sun

**Affiliations:** Liver Transplantation Center of the First Affiliated Hospital and State Key Laboratory of Reproductive Medicine, Nanjing Medical University, Nanjing, Jiangsu Province P.R. China; Department of Hematology, The First Affiliated Hospital of Nanjing Medical University, Nanjing, Jiangsu Province China; Liver Transplantation Center of the First Affiliated Hospital and State Key Laboratory of Reproductive Medicine, Nanjing Medical University, 300 Guangzhou Road, Nanjing, Jiangsu Province P.R. China

**Keywords:** ERα, MTA1, ER element, Corepressor, HCC

## Abstract

**Background:**

Although expression of MTA1 inversely correlates with the nuclear localization of ERα, the effect and molecular mechanism of ERα regulation of MTA1 remain unknown.

**Methods:**

Quantitative real-time PCR and western blot analyses were used to measure levels of MTA1. The effect on HCC cell proliferation and invasion was assessed by EdU incorporation assays and Transwell, respectively. ShRNA and dual-luciferase assays were used to investigate the regulatory relationship between MTA1 and ERα in cell lines.

**Results:**

We found that MTA1 gene regulation by ERα may be influenced by nuclear corepressors. The MTA1 promoter has three functional ER-element half-sites that lead to decreased MTA1 transcription and expression. ERα overexpression suppressed the proliferation and invasion of hepatocellular carcinoma cells (HCC). In addition, overexpression of MTA1 attenuated ERα-mediated suppression of the proliferation and invasion of HCC cells and tumor formation in vivo. These results suggested feedback regulation between ERα and MTA1. In summary, our results demonstrated that ERα suppressed proliferation and invasion of human HCC cells through downregulation of MTA1 transcription.

**Conclusions:**

Our study is an improved description of the mechanisms of the suppressive effect of ERα on HCCs, adding understanding to the gender disparity of HCC progression.

## Introduction

Many studies have explored the expression levels of MTA family members, especially MTA1, in human cancers [1]. MTA1 gene expression correlates with cancer progression and degree of invasion for hepatocellular carcinoma (HCC) and other carcinomas [2–5]. Of 20 HCC specimens with vascular invasion, 19 (95 %) displayed strong MTA1 expression. Overexpression of MTA1 also significantly correlates with large tumor size [6]. Increased MTA1 expression in HCC correlates with larger tumors, perinodal extension, and microvascular invasion [7]. High expression of the MTA1 gene is suggested to be a prognostic indicator after curative hepatectomy for HCC [2, 3]. Other data indicate that MTA1 is closely associated with microvascular invasion, frequent postoperative recurrence, and poor survival of HCC patients, especially those with HBV-associated HCC [7]. MTA1 is overexpressed in patients with invasive HBV-associated HCC. MTA1 overexpression is associated with shorter survival of patients with HBV-associated HCC after curative resection [8]. Previous reports also indicate that large tumors are more common in estrogen receptor (ER)-negative HCC. Furthermore, in a normal hepatocyte cell line, MTA1 expression was absent or minimal, whereas ERα was present in the nucleus [9]. Expression of MTA1 inversely correlates with ERα nuclear localization [6].

Several lines of evidence suggest involvement of sex hormones and their receptors in liver carcinogenesis. ERα is expressed in the liver of healthy individuals and patients with chronic hepatitis and HCC [10]. ER-positive HCC is malignant and has a better prognosis than ER-negative HCC [9]. HCC occurs more often in men than women by a ratio that ranges from 2:1 to 11:1 in several cohort reports [11]. Estrogen, which exerts its biological function through ERs, inhibits HBV replication [12]. A previous study showed that, in women, the risk of HCC is inversely related to age of menopause and number of full-term pregnancies [13]. This finding is consistent with animal studies showing that ovariectomy increases susceptibility to HCC in female mice [14]. ER is significantly downregulated in HCCs, both by immunohistochemistry staining and receptor-binding assays [15–18]. The steroid hormone 17 β-estradiol (E2) is important for controlling the expression of genes involved in a variety of biological processes including reproduction, development, and HCC progression. ERα is the major ER in the mammary epithelium. Ligand-activated ERα translocates to the nucleus, binds to estrogen response elements (EREs) in target gene promoters, and stimulates or represses gene transcription [19, 20]. The consensus ERE is a 13-bp palindromic sequence containing two inverted repeats of 5′-GGATC-3′ separated by three base pairs. ERα binds to nonperfect or half ERE sequences, particularly in the context of appropriate flanking sequences [21]. Corepressors are crucial regulators of ERα-mediated action and they might inhibit HCC development. An increasing number of ERα corepressors are being reported [22]. A previous study found that overexpression of ERα decreases PPARγ expression at the transcriptional and translational level in a ligand-dependent manner [23]. Among ER corepressors, NCoR and SMRT are widely characterized molecules implicated in transcriptional silencing in the absence of ligands [24]. Given the importance of ERα in HCC, the role of ERα corepressors in the molecular mechanism of ERα activity needs to be better understood.

In this study, we investigated the effects of ERα on three half-ERE sequences of the MTA1 promoter in HCC. Proliferation and invasion assays and animal model experiments provided evidence that inhibition of MTA1 by ERα is important for attenuating HCC progression. These findings are an improved characterization of the molecular events underlying the gender inconsistency of HCC progression.

## Materials and methods

### Ethics statement

All animal work was conducted under the institutional guidelines of Jiangsu Province and approved by the Use Committee for Animal Care.

### Chromatin immunoprecipitation

ChIP assays were performed by Pierce™ Agarose ChIP Kit (Thermo Fisher Scientific, Rockford, USA). HepG2 cells were treated with formaldehyde and incubated for 10 min to generate DNA-protein cross-links. Cell lysates were then sonicated to generate chromatin fragments and immunoprecipitated with ERα antibody or IgG as control. Precipitated chromatin DNA was recovered and analyzed by PCR. The PCR primers were: sense, 5′-CGCACGACCACCTGTCCA-3′, and anti-sense, 5′-GCCCTTCCACCAGAACCC-3′. The acquired DNA was resolved on a 2 % agarose gel and stained with Goldview.

### Real-time PCR

RNAs were extracted from cells using TRIzol (Invitrogen, California, USA) kit according to the manufacturer’s instructions. Subsequently, total RNA was reverse transcribed using SuperScript III reverse transcriptase (Invitrogen, California, USA). Real-time PCR was then performed in ABI PRISM7500 system (Applied Biosystems, California, USA), according to the manufacturer’s instructions. The expression level of each gene was normalized by GAPDH and reported as relative levels. The primers sequences for real-time PCR were obtain from the PrimerBank online. The PCR primers were: MTA1, sense, 5′-ACGCAACCCTGTCAGTCTG-3′, and anti-sense, 5′-GGGCAGGTCCACCATTTCC-3′; GAPDH, sense, 5′-GGAGCGAGATCCCTCCAAAAT-3′, and anti-sense, 5′- GGCTGTTGTCATACTTCTCATGG-3′.

(http://pga.mgh.harvard.edu/primerbank/).

### Dual luciferase reporter assay

The MTA1 gene −600 ~ −1 region was cloned into the pEZX-PG04 with Gaussia Luciferase (GLuc) and Secreted Alkaline Phosphatase (SEAP) labeled, named pEZX-PG04-MTA1 (GeneCopoeia, Guangzhou, China). For transient transfection, the plasmids were transfected into cells directly by lipofectamine 2000 (Invitrogen, USA). Secrete-Pair™ Dual Luminescence Assay kit (GeneCopoeia, Guangzhou, China) was performed 48 h after transfection according to the manufacturer’s protocol and detected with a Fluoroskan microplate reader (Thermo Labsystems, Helsinki, Finland). The activities of Gaussia Luciferase (GLuc) and Secreted Alkaline Phosphatase (SEAP) from cell culture medium were analyzed using a Secrete-Pair™ Dual Luminescence Assay kit according to the manufacturer’s instructions. Calculate the ratio of luminescence intensities (RLU, Relative Light Unit) of the GLuc over SEAP. Compare the normalized GLuc activity (GLuc/SEAP ratio) of all samples.

### Lentivirus production and infection

pLV-GFP vector system were constructed for overexpression of ERα and MTA1; pLL3.7-GFP vector system were constructed for ERα shRNA. The shRNA sequences were: 5′-AAC*GGCATGGAGCATCTCTACA*TTCAAGAGA*TGTAGAGATGCTCCATGCC*TTTTTTC-3′ [25] and 5′-AACTGGTTTACATGTCGACTAATTCAAGAGATTAGTCGACATGTAAACCTTTTTTTC-3′ for ERα and scramble, respectively. All constructs were sequence-verified. Details are available on request. Lentivirus of ERα, MTA1, and ERα shRNA were derived from our previous protocols [26]. The HCC cell lines HepG2 and Hep3B were obtained from Chinese Academy of Sciences and maintained in DMEM medium (Life Technologies, Carlsbad, CA, USA) supplemented with 10 % fetal bovine serum, 100 units/ml penicillin, and 100 μg/ml streptomycin (Invitrogen, Carlsbad, CA, USA) in a humidified 5 % CO2 incubator at 37 °C. The Hep3B-ERα, HepG2-shERα, and Hep3B-ERα/MTA1 cells were generated from our previous protocols [26].

### Western blotting

Whole cells were washed in PBS and lysed in RIPA lysis buffer supplemented with protease inhibitor cocktail (Roche, Mannheim, Germany). Total protein was quantified using a BCA Protein Assay Kit (Beyotime, Jiangsu, China), and an equal amount of whole cell lysates was resolved by SDS-polyacrylamide gel electrophoresis (PAGE) and transferred to a polyvinylidene difluoride (PVDF) membrane (Millipore, Eschborn, Germany). The blots were blocked in BSA (5 % w/v in PBS + 0.1 % Tween 20) for 1 h at room temperature. The following primary antibodies were used according to the manufacturer’s instructions. Antibodies against ERα, MTA1, and β-actin were purchased from Santa Cruz Biotechnology (Santa Cruz, CA). The appropriate secondary antibodies (Santa Cruz Biotechnology, Santa Cruz, CA, USA) were used at 1:1000–1:2000 (v/v) dilutions in PBS + 0.1 % Tween 20 for 1 h at room temperature, and the signals were revealed using ECL kit(Thermo Scientific Pierce, Thermo Fisher Scientific, Rockford, USA). The cells were induced with 10nM and 100 nM 17β-estradiol (E2) for 24 h.

### CCK-8 assay

Cell proliferation was measured by using the Cell Counting Kit-8 assay (CCK-8, Dojindo, Japan). Briefly, cells were plated into 96-well plates at a density of 10^4^ cells/well with 100 μL of culture medium. After adhesion the cells were incubated for 24, 48 and 72 h. At the end of each culture period, 10 μL CCK-8 reagent was added to each well and incubated for another 4 h, then the absorbance was measured at 450 nm wavelength.

### EdU assay

Cells (2 × 10^3^ cells/well) were seeded in triplicate in 96-well plates and incubated overnight. Cells were starved in DMEM without FBS for 24 h and then incubated in DMEM containing 5 % FBS. Then the EdU (5′-ethynyl-2′-deoxyuridine) incorporation assay was performed to quantify cell proliferation using the Cell-Light™ EdU DNA Cell Proliferation Kit (Guangzhou Ribobio Co., Ltd, Guangzhou, China) according to the manufacturer’s instructions. More than five random fields per well were captured, and IPP 6.0 was used to calculate the percentage of EdU-positive cells in total cells.

### Transwell invasion assay

Cell invasion assays were evaluated using Transwell cell migration plates (Corning, NY, USA) and 8-μm pore size Matrigel invasion chambers (BD Biosciences, San Jose, USA) according to the manufacturer’s instructions [23–27]. Cells (1.0 × 10^4^) were seeded in serum-free medium into the upper chamber and allowed to invade towards 10 % FBS in the lower chamber. After 24 h incubation in 37 °C and 5 % CO_2_, the cells invaded through the membrane and adhered to the underside of the membrane. Then cells were fixed and stained with crystal violet. The images were acquired by using NIS Elements image analysis software (Nikon, Tokyo, Japan). For the membrane images, we measure the migrated cells using image analysis software ImagePro Plus 6.0 (Media Cybernetics, Bethesda, USA).

### Mice model

Nude mice were purchased from the Vital River Laboratories (Beijing, China). All animals were used in accordance with institutional guidelines and the current experiments were approved by the Use Committee for Animal Care. All cultures used for injection were sub-confluent and were fed the day prior to use. The harvested cell suspension was washed twice by centrifugation in medium containing serum at room temperature and then resuspended in medium without serum at 4 °C immediately prior to injection. The number of cells (1 × 10^5^ cells) to be injected was suspended in 0.1 ml PBS. The cells were were injected subcutaneously (into groin). The animals were sacrificed 4 weeks after injection. Pictures were recorded with a Nikon d800 digital camera (Nikon, Tokyo, Japan).

### Statistical analysis

All results were expressed as the mean ± s.d. The Student’s t-tests were used to analyse significant differences between samples. All the histogram was evaluated by performing GraphPad Prism, version 4.0 (GraphPad Software, San Diego California, USA). Statistical analyses were performed using Stata. 11.0. *P* <0.05 indicated statistically significant.

## Results

### ERα binds the MTA1 promoter and downregulates MTA1 expression

**Fig. 1:**
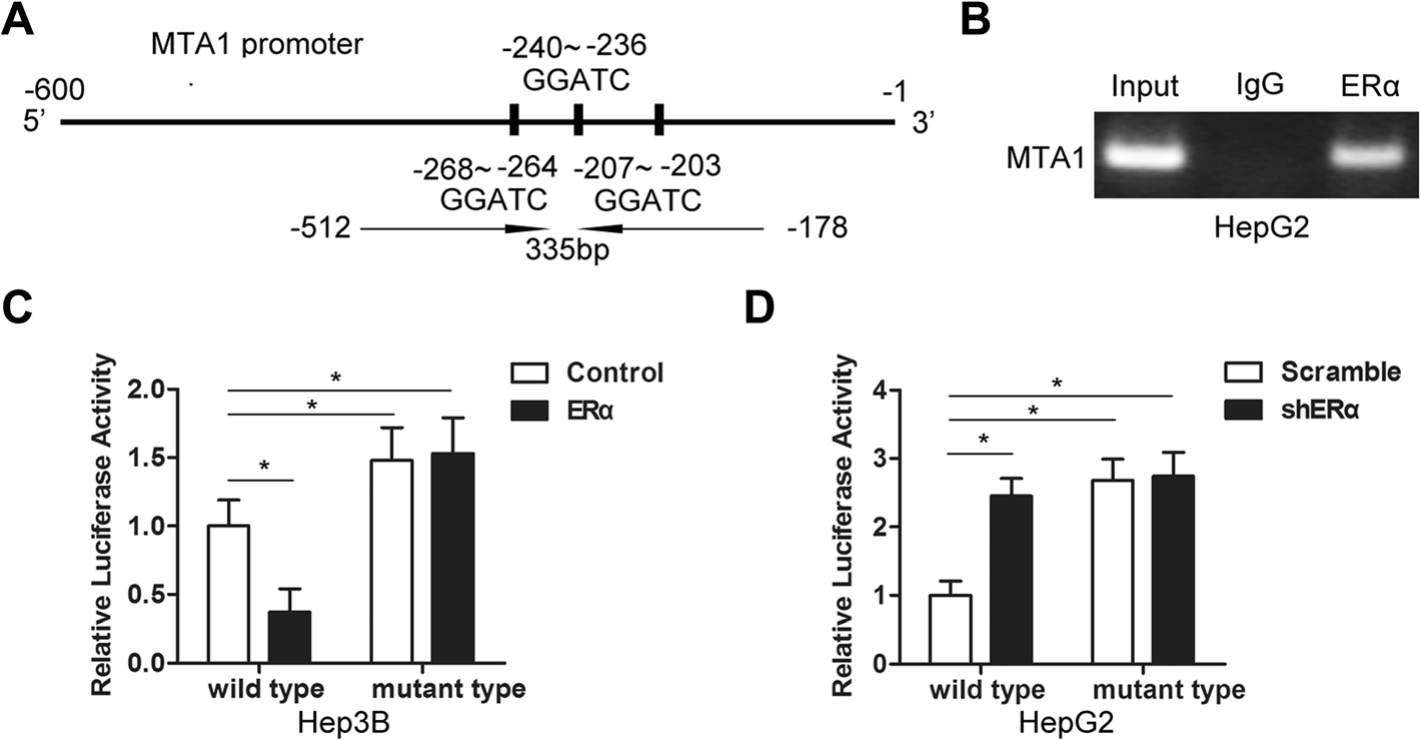
ERα bound to the MTA1 promoter and downregulated MTA1 expression. **a** Schematic representation of the MTA1 promoter region with three half-ERE sites. Specific primers amplified –512 to −178 by PCR. **b** Chromatin immunoprecipitation (ChIP) assays used normal IgG or anti-ERα to identify ERα binding sites on the MTA1 promoter in HepG2 cells. PCR product was 335 base pairs. Three half-ERE sites were deleted from the wildtype MTA1 promoter for the mutant MTA1 promoter. Hep3B (**c**) and HepG2 (**d**) cells were cotransfected with luciferase vectors with wildtype or mutant MTA1 promoter or control vector. Results were from three independent experiments and presented as mean ± SEM. *P < 0.05 by *t*-test

**Fig. 2:**
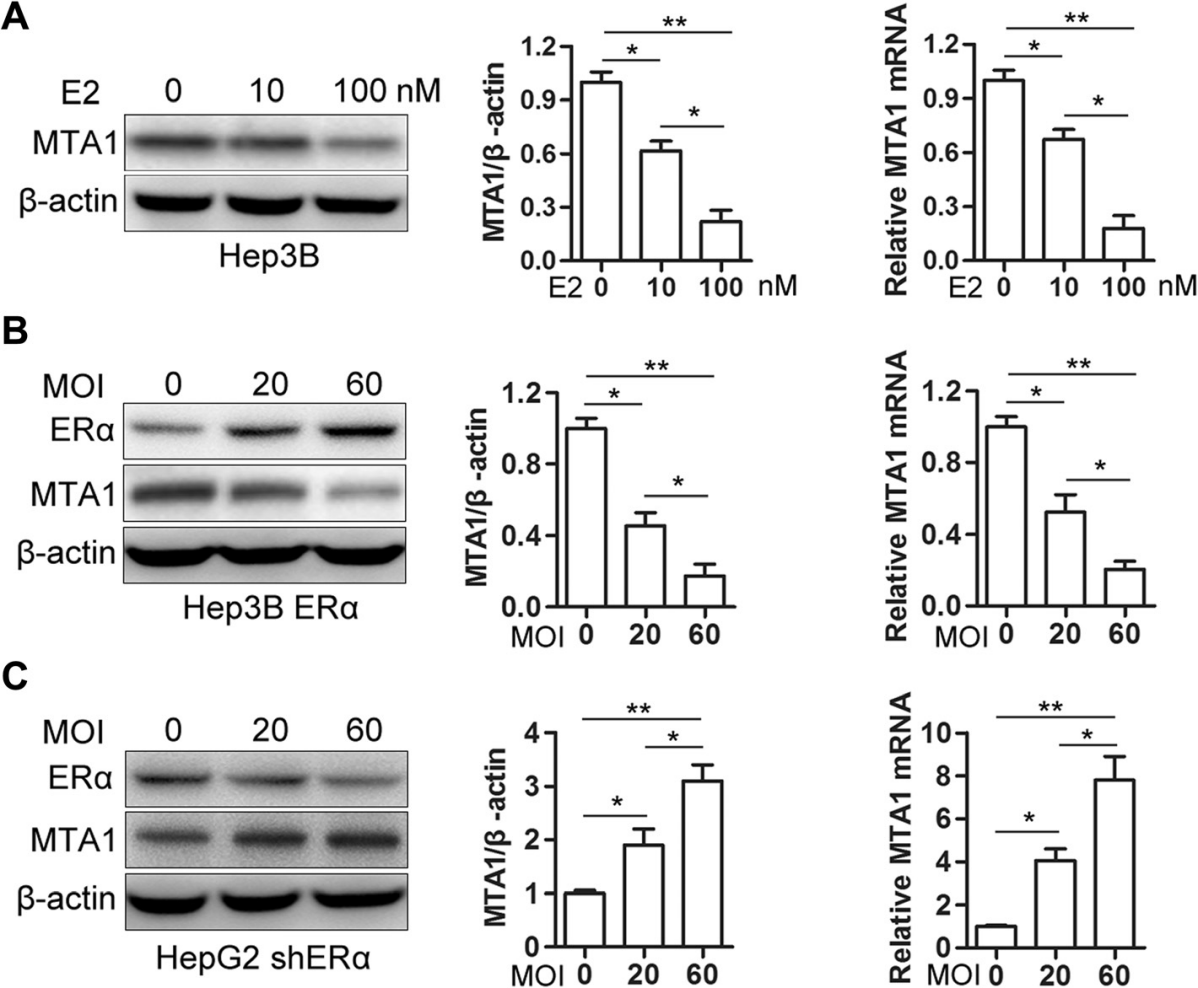
ERα decreased MTA1 expression in HCC cells. **a** Hep3B cells were maintained in phenol red-free DMEM with 10 % dextran-coated charcoal-treated FBS for 48 h and cells treated with either ethanol vehicle or E2 (10 or 100 nM) for 72 h. Cells were harvested and analyzed for MTA1 mRNA (*right*) and protein. Results were from three independent experiments and presented as mean ± SEM. **b** Western blot and real-time PCR analysis (*right*) for MTA1 protein and mRNA in Hep3B cells with MOI 20 or 60 ERα lentivirus infection. Results were from three independent experiments and presented as mean ± SEM. **c** Western blot and real-time PCR analysis (*right*) for MTA1 protein and mRNA in HepG2 cells with MOI 20 or 60 for shRNA-ERα lentivirus infection (*n* = 3, mean ± SEM) *P < 0.05, **P < 0.01 by *t*-test

Previous studies show that expression of MTA1 inversely correlates with ERα nuclear localization [6]. We searched for EREs within the BMI1 promoter. We found three half-ERE sites from −268 to −203 (Fig. 1a). To explore whether ERα assembled a complex on the MTA1 promoter, we performed chromatin immunoprecipitation (ChIP) assays. ERα bound to the region between −512 to −178 that contained the three half-ERE sites (Fig. 2b). To study the regulation of the MTA1 promoter via the ERE half-sites, we generated a wildtype MTA1 promoter fragment and a mutant MTA1 promoter with a deletion from −600 to −1 including the three ERE half-sites. The MTA1 promoter fragments were cloned into a pEZX-PG04 luciferase reporter system. The luciferase activity of wildtype and mutant type MTA1 promoter-luciferase vectors was tested in Hep3B and HepG2 cells. Ectopic overexpression of ERα significantly decreased MTA1 promoter activity in Hep3B cells transfected with the wildtype MTA1 promoter vector but had no effect on cells transfected with the mutant MTA1 promoter vector (Fig. 1c). Transfection with ERα shRNA increased MTA1 promoter activity in HepG2 cells but did not have an effect on the mutant MTA1 promoter (Fig. 1d). Together, these findings indicated that recruitment of ERα to MTA1 promoter chromatin was accompanied by repressed MTA1 promoter activity.

### ERα downregulated MTA1 expression in HCC cells

Hep3B cells were cultured in estrogen-free medium and treated with various concentrations of E2 for 72 h. With E2, MTA1 protein and mRNA decreased (Fig. 2a). Infecting Hep3B cells with an ERα lentivirus at different MOIs resulted in reduction of MTA1 protein and mRNA with increasing lentivirus MOI (Fig. 2b). To further investigate the regulatory effect of ERα, we knocked down ERα in HepG2 cells using shRNA lentivirus infection and observed MTA1 expression. As shown in Fig. 2c, knockdown of endogenous ERα significantly increased MTA1 protein and mRNA in a dose-dependent manner.

### ERα suppressed the proliferation and invasion of HCC cells

**Fig. 3:**
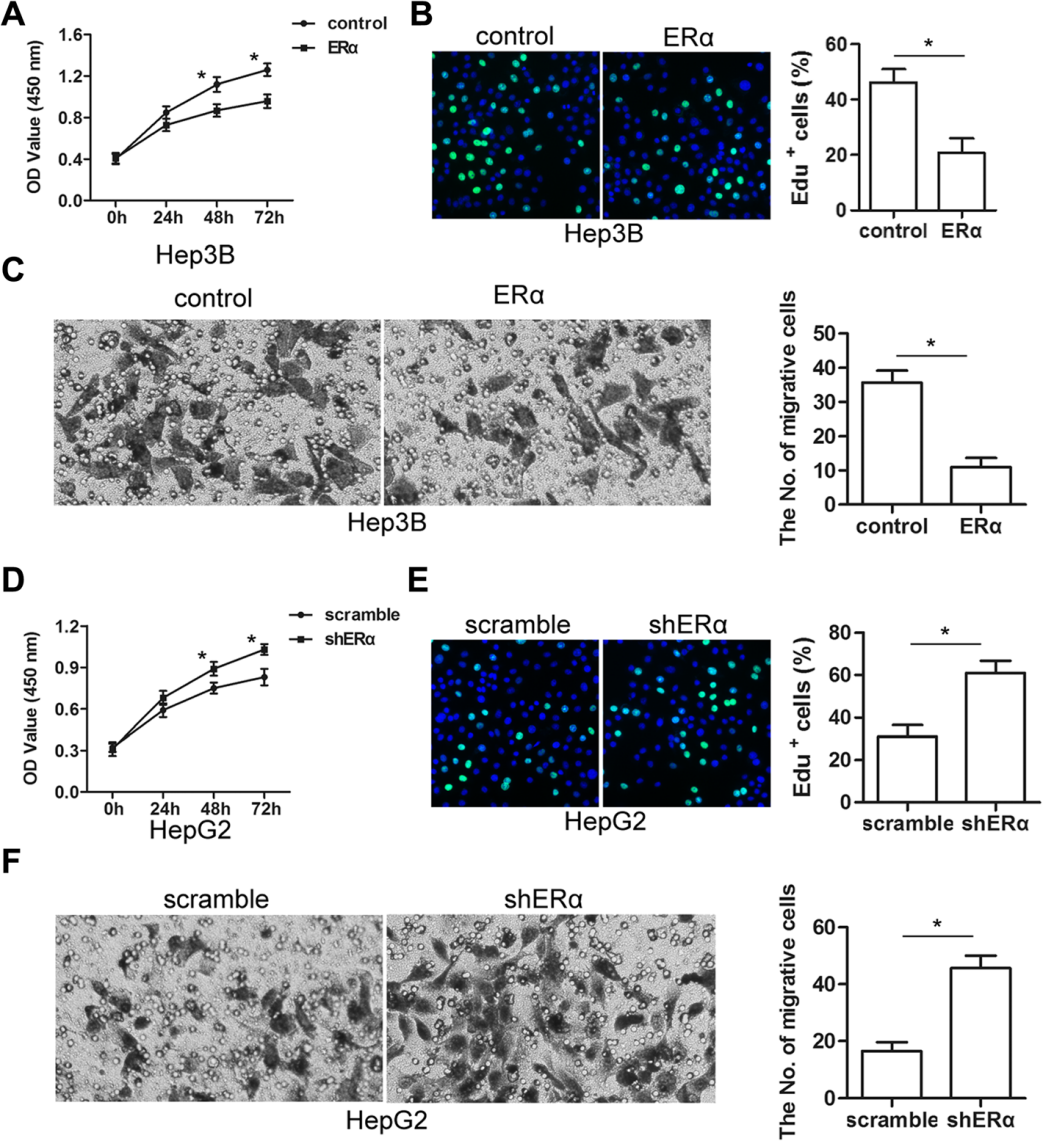
ERα suppressed proliferation and invasion of HCC cells. **a** CCK-8 assays showed that overexpression of ERα inhibited Hep3B cell growth. Absorbance at 450 nm was measured. Results were from three independent experiments and presented as mean ± SEM. **b** Bright cyan, EdU positive. EdU assays showed that ERα overexpression decreased the percent of EdU-positive in Hep3B cells compared to controls (*n* = 3, mean ± SEM). **c** Overexpression of ERα reduced invasion by Hep3B cells in transwell assays (*n* = 3, mean ± SEM). **d** CCK-8 assays showed that knockdown of ERα increased HepG2 cell growth (*n* = 3, mean ± SEM). **e** Bright cyan, EdU-positive. EdU assays showed that ERα knockdown increased the percent of EdU-positive HepG2 cells compared to controls (*n* = 3, mean ± SEM). **f** Knockdown of ERα increased invasion by HepG2 cells in transwell assays (*n* = 3, mean ± SEM) *P < 0.05 by *t*-test

Based on these results, we hypothesized that the proliferation and invasion effects of MTA1 would be suppressed by ERα in HCC cells. To determine if ERα overexpression suppressed proliferation and invasion, we constructed the cell line Hep3B-ERα, which overexpressed ERα. CCK-8 proliferation assays revealed that proliferation of Hep3B-ERα cells was significantly reduced compared to control Hep3B-vector cells (Fig. 3a). To verify this result, we used 5-ethynyl-2′-deoxyuridine (EdU) in dynamic proliferation assays. Overexpression of ERα impaired the proliferation of Hep3B cells (Fig. 3b). Because MTA1 enhances the invasion of HCC, we used transwell invasion assays to examine the effect of ERα on invasion. The invasive capacity of Hep3B-ERα cells was significantly lower than control cells (Fig. 3c). Specific shRNA targeting ERα knocked down endogenous ERα in HepG2 cells. ERα knockdown increased the proliferation of HepG2-shERα cells compared to control scramble-shRNA-treated cells (Fig. 3d). EdU assays indicated that ERα knockdown increased cell proliferation of HepG2-shERα cells (Fig. 3e). Matrigel invasion assays also demonstrated that ablation of endogenous ERα increased invasion by HepG2-shERα cells (Fig. 3f). Collectively, these results provided evidence that ERα expression was important for the aggressive phenotype of HCC cells.

### Reestablishment of elevated MTA1 by ectopic expression abrogated ERα-mediated suppression of proliferation and invasion

**Fig. 4:**
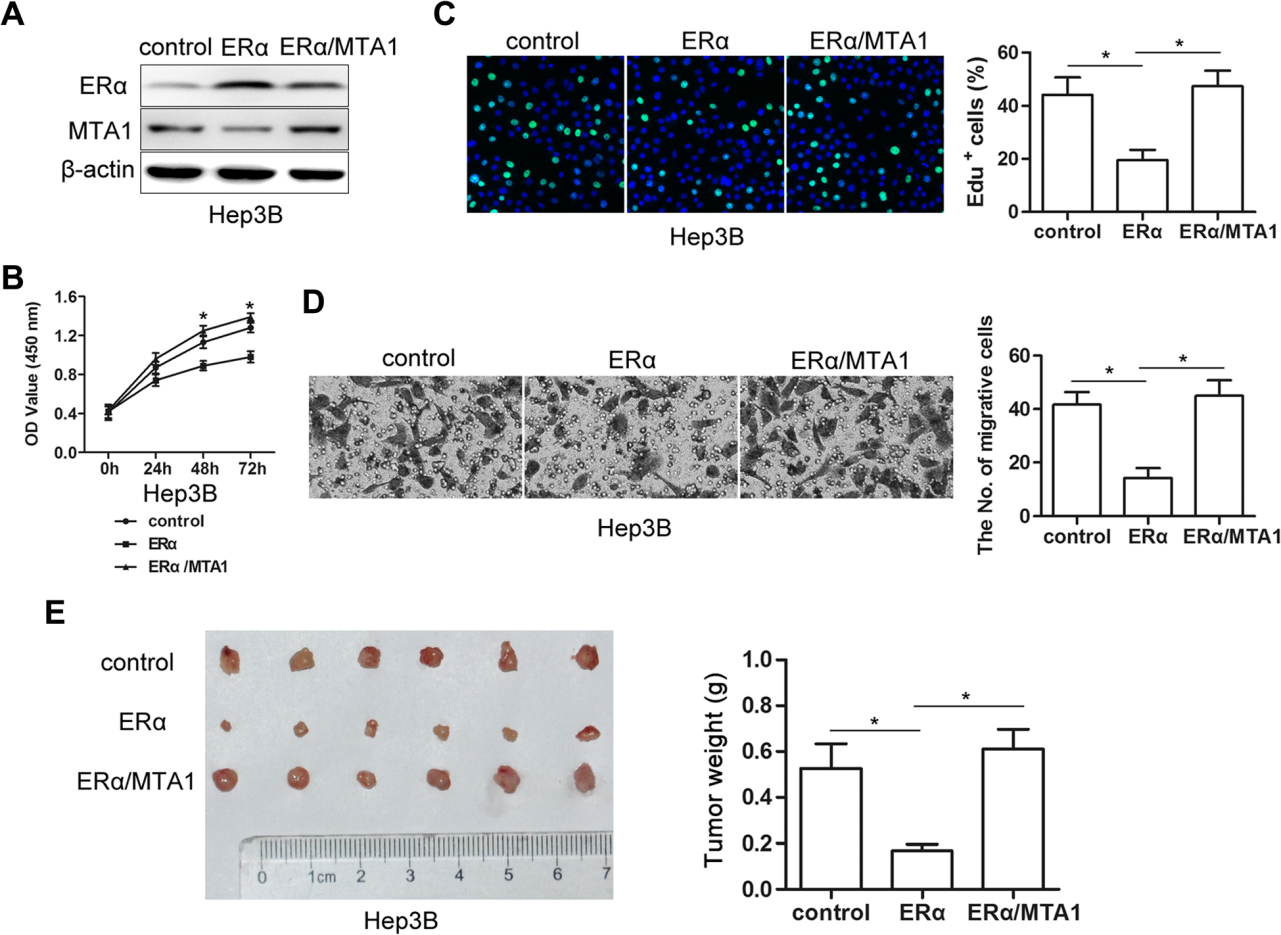
Restoration of elevated MTA1 by ectopic expression abrogated ERα-mediated suppression of proliferation and invasion. **a** Western blots showed that Hep3B-ERα/MTA1 cells exhibited ERα and MTA1 ectopic expression after ERα or MTA1 lentivirus infection. **b** MTA1 overexpression increased growth of Hep3B-ERα/MTA1 compared to Hep3B-ERα cells. Absorbance at 450 nm was measured. Results were from three independent experiments and presented as mean ± SEM. **c** Bright cyan, EdU-positive. EdU assays showed that restoration of MTA1 increased the percent of EdU-positive cells compared to Hep3B-ERα cells (*n* = 3, mean ± SEM). **d** Representative images of transwell invasion assays. MTA1 overexpression increased invasion by Hep3B-ERα/MTA1 cells compared to Hep3B-ERα cells (*n* = 3, mean ± SEM). **e** Macroscopic images (*left*) and weights of subcutaneous tumors (*right*) on day 28 after s.c.-administered Hep3B-ERα/MTA1, Hep3B-ERα and control cells. Data are mean ± SEM of tumor weights (*n* = 6 each). *P < 0.05 by *t*-test

MTA1 protein decreased in Hep3B-ERα cell lines in which ERα was ectopically overexpressed. To determine if ERα suppressed proliferation and invasion by reducing MTA1 expression, we constructed the cell line Hep3B-ERα/MTA1, which expressed ERα and ectopically overexpressed MTA1. Western blots showed that overexpression of MTA1 decreased expression of ERα in some Hep3B-ERα/MTA1 cells compared to Hep3B-ERα cells (Fig. 4a) with feedback regulation between ERα and MTA1. CCK-8 and EdU assays demonstrated that MTA1 overexpression increased proliferation of Hep3B-ERα cells (Fig. 4b and c). Matrigel invasion assays showed that invasion of Hep3B-ERα cells significantly increased (Fig. 4d).

To test whether ERα overexpression decreased tumor formation by Hep3B cells, a subcutaneous injection model was established in BALB/c nude mice. At 4 weeks after injection of tumor cells, mice injected with Hep3B-ERα cells showed decreased tumor sizes compare to mice injected with Hep3B cells (Fig. 4e). Restoration of MTA1 expression resulted in significantly increased tumor sizes in Hep3B-ERα/MTA1 (Fig. 4e). These findings suggested that inhibition of MTA1 function by ERα impaired HCC proliferation and invasion.

## Discussion

Previous studies on gender disparities in HCC highlight the linkage between oestrogen and inflammation-induced carcinogenesis [28]. Physiological doses of estrogen suppress HCC metastasis by decreasing interleukin-6 and hepatocyte growth factor expression in the tumor microenvironment [29]. MiR-18a prevents translation of ER, potentially blocking the protective effects of estrogen and promoting the development of HCC in women [11]. Elevated p53 promotes miR-18a processing to decrease ERα in HCC in women [30]. Oestrogen modulates HCC malignancy in vivo by reducing tumor cell invasion, arresting cell cycle progression, and promoting apoptosis, characterized by decreased expression of MMP-2, MMP-9, PCNA, cyclin A, cyclin D1, and Bcl-2, and increased expression of cleaved caspase 3. ERα-mediated inhibition of NF-kappaB binding is a pivotal event [23, 31]. Consistent with recent reports, we found that overexpression of ERα suppressed HCC proliferation and invasion and decreased MTA1 expression. In our previous study, we found that PTPRO expression results in pathological deficiency and gender bias in HCC, which could be attributed to ERα regulation [26]. Overexpression of miR-22 in male tumor adjacent tissue was associated with downregulated ERα expression, potentially by attenuating the protective effect of estrogen and causing increased IL-1α expression [32] and STAT3 and IL-1α involved in inflammation and stemness inducible of HCC progression. These mechanisms might be responsible of the in vivo effect of ERa on reduction of tumor growth in this study which could not be fully appreciated in the in vitro experiments on cell proliferation.

Transcriptional activation by ERα is a complex and multistep process that is influenced by coactivator and corepressor proteins that either positively or negatively modulate ERα-mediated transcriptional activity [21, 33]. The transcriptional activity of ERα is modified by coactivators, corepressors, and chromatin remodeling complexes. In our study, ERα was recruited to the MTA1 promoter chromatin to repress MTA1 promoter activity and downregulate MTA1 expression. Different ERα corepressors regulate steroid receptor activity through a variety of mechanisms, including formation of multiprotein complexes that affect chromatin remodeling, histone deacetylation, or basal transcription [21]. Transcriptional repression, which is crucial for diverse biological processes, is mediated in part by non-DNA-binding corepressors [19]. Transcriptional repression by tamoxifen-bound ER at E-regulated gene promoters involves a dynamic interplay of multiple distinct chromatin-modifying and remodeling complexes [34]. Overexpression of ERα decreases PPARγ expression at the transcriptional and translational level in a ligand-dependent manner [35]. We hypothesized that expression of MTA1 would be determined by the level of ERα. Additionally, the ERα-SP1 complex binds to the proximal and distal sites of the TNFα gene promoter and induces expression of active caspase three in a ligand-dependent manner [36]. ERα inhibits the epithelial-mesenchymal transition by suppressing Bmi1 in breast cancer [37]. The N-terminus of NCOR2 interacts with other transcriptional corepressors to regulate transcription and its C-terminus binds to ERα [38]. The transcriptional corepressor TLE3 is recruited to chromatin by FoxA1 at several ER-binding sites throughout the genome. TLE3 mediates repression via interaction with HDACs to regulate histone acetylation [39]. Foxa factors have a dominant effect on gender specificity in HCC development, while Foxa-dependent ERα and AR have opposing effects in HCC, with ERα-mediated estrogen signaling protective against HCC [40]. One of these mechanisms could be involved in regulation of MTA1.

MTA1 and MTA1s are corepressor proteins that act in different ways to prevent ERα transcriptional activity [41]. The shRNA targeted against MTA1 could specifically mediate the MTA1 gene silence and consequentially recover the protein expression of ERα [42]. MTA1-TFAP2C or the MTA1-IFI16 complex may contribute to the epigenetic regulation of ERα expression in breast cancer [43]. MTA1 and MICoA might transmodulate each other’s functions. Any potential deregulation of MTA1 is likely to contribute to the functional inactivation of the ER pathway, presumably by derecruitment of MICoA from chromatin with ER promoter targets [44]. The MTA3 promoter has a functional ER element half-site and MTA3 gene regulation by ER is influenced by dynamic changes in levels of nuclear coregulators, including MTA1 [45]. Overexpression of MTA1 resulted in a few Hep3B-ERα/MTA1 cells with decreased expression of ERα compared to Hep3B-ERα cell (Fig. 4a). Based on these results, feedback regulation between ERα and MTA1 might occur. This will be studied in our further research.

## Conclusions

Our results showed that ERα suppressed proliferation and invasion of human HCC cells through transcriptional downregulation of MTA1. Our study is an improved description of the mechanisms of the suppressive effects of ERα on HCC and adds understanding to the gender disparity in HCC progression.

## Abbreviations

GAPDH: Glyceraldehyde 3-phosphate dehydrogenase
HCC: Hepatocellular carcinoma
lncRNA: Long non-coding RNAs
qRT-PCR: Quantitative real time polymerase chain reaction
SDS-PAGE: Sodium dodecyl sulphate polyacrylamide gel electrophoresis
10 % FBS: 10 % fetal bovine serum
EDU: 5-ethynyl-2′-deoxyuridine
