# Correction: Inhibition of MTA1 by ERα contributes to protection hepatocellular carcinoma from tumor proliferation and metastasis

**DOI:** 10.1186/s13046-024-03091-y

**Published:** 2024-06-19

**Authors:** Lei Deng, Hui Yang, Junwei Tang, Zhe Lin, Aihong Yin, Yun Gao, Xuehao Wang, Runqiu Jiang, Beicheng Sun

**Affiliations:** 1 Liver Transplantation Center of the First Affiliated Hospital and State Key Laboratory of Reproductive Medicine, Nanjing Medical University, Nanjing, Jiangsu Province People’s Republic of China; 2 Department of Hematology, The First Affiliated Hospital of Nanjing Medical University, Nanjing, Jiangsu Province China

**Correction: J Exp Clin Cancer Res 34, 128 (2015)**

**https://doi.org/10.1186/s13046-015-0248-0**

Following publication of the original article [1], authors identified an error in Fig. 4D (left and middle panel). The original image was mistakenly used and did not correspond to the experimental data described.

The correction does not affect the overall result or conclusion of the article. The original article [1] has been corrected.

**Incorrect Figure 4**

**Fig. 4:**
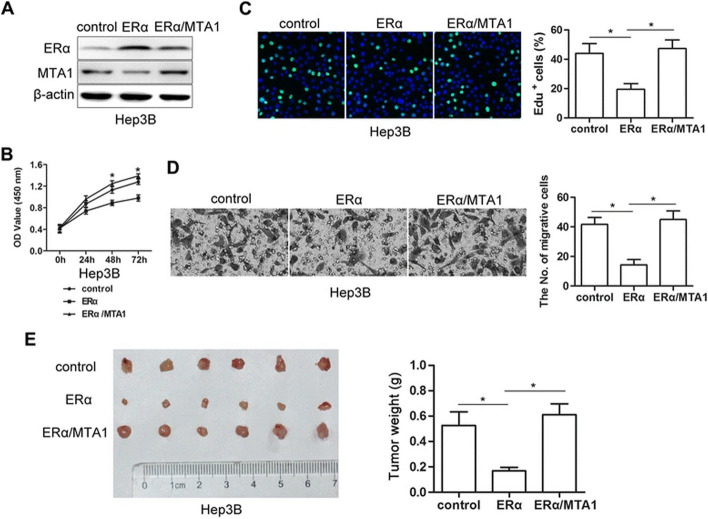
Restoration of elevated MTA1 by ectopic expression abrogated ERα-mediated suppression of proliferation and invasion. **a** Western blots showed that Hep3B-ERα/MTA1 cells exhibited ERα and MTA1 ectopic expression after ERα or MTA1 lentivirus infection. **b** MTA1 overexpression increased growth of Hep3B-ERα/MTA1 compared to Hep3B-ERα cells. Absorbance at 450 nm was measured. Results were from three independent experiments and presented as mean ± SEM. **c** Bright cyan, EdU-positive. EdU assays showed that restoration of MTA1 increased the percent of EdU-positive cells compared to Hep3B-ERα cells (*n* = 3, mean ± SEM). **d** Representative images of transwell invasion assays. MTA1 overexpression increased invasion by Hep3B-ERα/MTA1 cells compared to Hep3B-ERα cells (n = 3, mean ± SEM). **e** Macroscopic images (*left*) and weights of subcutaneous tumors (*right*) on day 28 after s.c.-administered Hep3B-ERα/MTA1, Hep3B-ERα and control cells. Data are mean ± SEM of tumor weights (*n* = 6 each). *P < 0.05 by t-test

**Correct Figure 4**

**Fig. 4:**
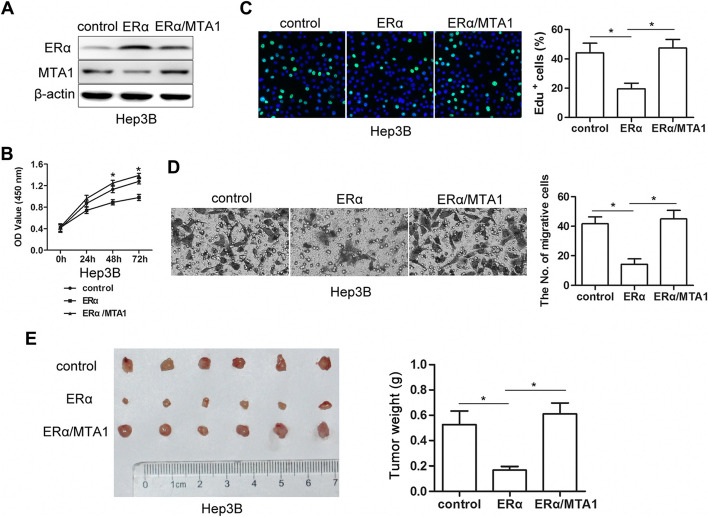
Restoration of elevated MTA1 by ectopic expression abrogated ERα-mediated suppression of proliferation and invasion. **a** Western blots showed that Hep3B-ERα/MTA1 cells exhibited ERα and MTA1 ectopic expression after ERα or MTA1 lentivirus infection. **b** MTA1 overexpression increased growth of Hep3B-ERα/MTA1 compared to Hep3B-ERα cells. Absorbance at 450 nm was measured. Results were from three independent experiments and presented as mean ± SEM. **c** Bright cyan, EdU-positive. EdU assays showed that restoration of MTA1 increased the percent of EdU-positive cells compared to Hep3B-ERα cells (*n* = 3, mean ± SEM). **d** Representative images of transwell invasion assays. MTA1 overexpression increased invasion by Hep3B-ERα/MTA1 cells compared to Hep3B-ERα cells (n = 3, mean ± SEM). **e** Macroscopic images (*left*) and weights of subcutaneous tumors (*right*) on day 28 after s.c.-administered Hep3B-ERα/MTA1, Hep3B-ERα and control cells. Data are mean ± SEM of tumor weights (*n* = 6 each). *P < 0.05 by t-test
